# Effects of Extreme Climatic Events on Evolutionary Processes

**DOI:** 10.1111/gcb.70618

**Published:** 2025-11-25

**Authors:** Robin Heinen, Madhav Prakash Thakur, Jeffrey Alan Harvey, Rieta Gols

**Affiliations:** ^1^ Terrestrial Ecology Research Group, School of Life Sciences Technical University of Munich Freising Germany; ^2^ Institute of Ecology and Evolution University of Bern Bern Switzerland; ^3^ Netherlands Institute of Ecology Wageningen the Netherlands; ^4^ Department of Ecological Sciences – Animal Ecology Vrije Universiteit Amsterdam Amsterdam the Netherlands; ^5^ Laboratory of Entomology, Wageningen University & Research Wageningen the Netherlands

**Keywords:** climate change, extreme temperature, flooding, gene flow, genetic diversity, genetic drift, heatwave, mutation, natural selection, wildfire

## Abstract

Climate change, through its vast impacts on biodiversity, is one of the most‐studied drivers of ecological change. Although reports of detrimental impacts of gradual global warming on the behavior and physiology of individuals, as well as on populations and communities are now common in scientific literature, much less is known about the impact of extreme climatic events (ECEs) on evolutionary processes. In this review, we provide a broad overview of the state of knowledge on ECEs in the context of evolution. We begin by discussing the drivers of evolution (i.e., mutation, selection, gene flow, and genetic drift) and how ECEs may impact them. We then discuss why rapid adaptation and evolutionary rescue in response to ECEs will be hindered in many species, due to the unpredictable nature and timing of these events. We further outline that potential changes in evolutionary processes in response to ECEs can be better understood by recognizing shifts in ecological interactions, emphasizing the connected nature of communities and ecosystems and the evolutionary consequences. We finally highlight that there is a clear gap in our knowledge of ECE impacts, particularly at the genetic level. In order to understand the relationships between climate change, ECEs, and evolutionary processes, we urgently need hypothesis‐driven monitoring efforts and studies that investigate existing data through the lens of historically documented ECE events. Taken together, our review highlights that extreme climatic events associated with climate change are undermining biodiversity through diverse pathways, and that the prospects for rapid adaptation and evolutionary rescue are severely constrained by a host of ecological and genetic challenges.

## Graphical abstract

Climate extremes are posing highly variable risks to species across the globe. Whether species will be able to evolutionarily adapt to such forces is not well‐understood. We review the evidence for evolutionary adaptations to such extremes and discuss the importance of ecological context.

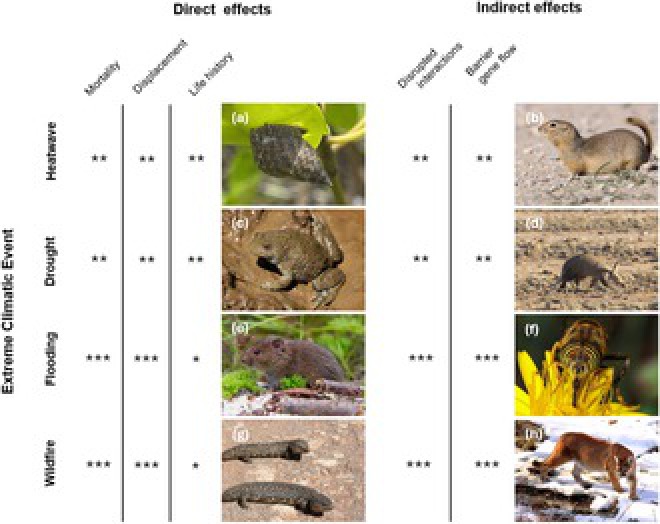

## 1 Introduction

Climate change is defined by the Intergovernmental Panel on Climate Change as a change in the state of the climate that can be identified statistically by variations in mean and variability of its properties and usually persists for extended periods of decades or more (IPCC 2023). The effect of human behavior on climate change seen over the past century, that is, anthropogenic climate change (ACC) has seen no parallel in the present geological epoch, the Holocene, and therefore poses a unique and novel threat to most extant species. During earlier planetary warming events human impacts on the biosphere were negligible or absent entirely. The planet's surface was less fragmented and simplified by large agricultural or urban expanses, and climatic refugia were likely larger, more numerous and more closely connected than they are under recent anthropogenic warming (Opdam and Wascher 2004; McGuire et al. 2016; Holyoak and Heath 2016; Frazier et al. 2025), indicating that human presence poses unique challenges to biodiversity.

Indeed, ACC has dramatic and mostly negative consequences on biodiversity (Araujo and Rahbek 2006; Bellard et al. 2012; Urban et al. 2016; Palmer et al. 2017). ACC affects the distribution of organisms, often resulting in species shifting their geographic ranges towards higher latitudes and altitudes to maintain suitable climatic niches (Thomas 2010; MacLean and Beissinger 2017; Chapman et al. 2015). ACC also has strong impacts on seasonal phenology (Kingsolver and Buckley 2018; Inouye 2022), particularly in a multitrophic context, where phenological mismatches commonly occur due to differing responses among interacting organisms in different trophic levels (Renner and Zohner 2018; Damien and Tougeron 2019; Visser and Gienapp 2019). Disproportionate effects of climate change in different trophic levels may disrupt interactions between producers and consumers leading to population declines that threaten food web structure and ecosystem functioning (Rosenblatt and Schmitz 2016; Bartley et al. 2019). Finally, ACC affects the physiology of species at higher temperatures, such as through shifts in body size, to adjust for increased metabolic demands (Savage et al. 2004; Gardner et al. 2011; Lindmark et al. 2018; Verberk et al. 2021). Taken together, it is evident that ACC through gradual deterministic warming alters biodiversity by affecting the distribution, phenology and physiology of organisms. However, the evolutionary consequences particularly from stochastic elements of ACC, including climatic extremes, are currently poorly understood.

The gradual rise in surface temperatures over decades across much of the biosphere represents only one aspect of ACC (IPCC 2023). Another aspect, occurring at even smaller temporal and spatial scales than gradual warming, that is, taking place within the span of days to weeks, is extreme climatic events (ECEs). These may include heatwaves, droughts and intense cloudbursts, and although ECEs are not unique to our times, research shows that they are increasing in their frequency, intensity and duration in many regions (Christidis et al. 2015; Mazdiyasni and AghaKouchak 2015; Meehl and Tebaldi 2004; Palmer 2014). In this review, we consider ECEs in the broadest sense, including rare climatic events (frequency), intense climatic events (threshold exceedance), or impactful events (environmental cost; see Beniston and Stephenson (2004)). In contrast to gradual climatic change, ECEs such as extreme heat may (temporarily) push species beyond critical physiological thresholds with potentially devastating consequences in terms of survival and reproduction (Moreno and Møller 2011; Colinet et al. 2015; Buckley and Kingsolver 2021). Numerous studies have shown that ECEs can impact populations and thus ripple up to affect the ecological dynamics of food webs involving multiple species (Bailey and van de Pol 2016; Harvey et al. 2020, 2023). Despite many studies showing direct negative consequences of long and short‐term exposure to ACC and ECEs, the strength and directions of these impacts may vary among taxa (Thakur et al. 2022; Gu et al. 2023). Although it is evident that many species of plants animals, and their habitats, are highly vulnerable to ECEs (Harris et al. 2018; Prugh et al. 2018; Trisos et al. 2020), the long‐term (evolutionary) consequences of such abrupt events on biodiversity remain unclear and thus require further investigation (Davis et al. 2005; Cinto Mejía and Wetzel 2023; Rising et al. 2022; Thakur et al. 2022).

Elucidating the larger‐scale effects of ECEs on communities and ecosystems requires a broader understanding of evolutionary responses to them among individuals and populations. Importantly, whereas ecologists traditionally focus on immediate responses to abiotic stressors, evolutionary biologists generally focus on longer‐term responses. Reciprocally, long‐term evolutionary responses to ECEs are likely to depend partly on immediate responses among organisms (Martínez‐De León and Thakur 2024). Thus far, however, ECE research has been dominated by short‐term studies, whereas fewer studies have examined long‐term effects (Bailey and van de Pol 2016; Thakur et al. 2022). These different approaches urgently need to be more effectively bridged to better understand and predict the impact of ECEs on biodiversity and community structure.

Here, we discuss the evolutionary consequences of ECEs in organisms and use this information to discuss long‐term biodiversity scenarios that may therefore emerge from them. We open with a brief presentation of the four main drivers of evolution and how exposure to ECEs may directly or indirectly interfere with them. This lays the foundation for one of the central messages in this article: that exposure to ECEs broadly has negative effects on biodiversity and thus reduces the resilience and stability of food webs and communities. A recent paper by Zhou and Wang (2023) proposed mechanisms that can negate the negative consequences of exposure to ECEs at various levels of biological organisation (i.e., from the individual to communities). The authors argued that stress‐induced responses associated with ECEs can even benefit species by selecting for genotypes that can cope with them, thus having long‐term evolutionary benefits. We challenge this idea and argue that exposure to ECEs is more likely to weaken the ability of species to persist or adapt to future ECEs.

## 2 Drivers of Evolution in a Changing World

Genetic diversity lies at the basis of the evolutionary principle. Four processes are often considered important drivers of evolution: mutation, natural selection, gene flow, and genetic drift. ECEs may both directly and indirectly interfere with these processes.

### 2.1 Mutation and Climate Change

Mutation is the process by which new genetic variation can enter a species' genetic pool either through spontaneous substitution of nucleotides, or through deletion, insertion or rearrangement of sections of DNA during replication. It has been well‐documented that mutation rates strongly differ between taxa (Allio et al. 2017) and among different genotypes of the same species (Haag‐Liautard et al. 2007). Mutation rates are slower in bacteria and archaea than in multicellular organisms (Agrawal and Whitlock 2010), yet they decrease with genome size in viruses and prokaryotes, whereas this relationship is reversed in eukaryotes (Lynch 2010). Moreover, evolutionary trajectories may differ depending on ploidy, that is, the number of homologous chromosome pairs per cell, and between sexual and asexual reproducing organisms due to differences in mutation rates and efficacy of selection (the rate at which a beneficial mutation becomes fixed in the population) (Orr and Otto 1994; Gerstein 2013). Spontaneous mutations can have deleterious, neutral or beneficial fitness consequences. To determine the relative frequencies of these types of mutations is difficult as the status of the mutation can vary depending on environmental conditions, especially when these conditions are extreme (Agrawal and Whitlock 2012). Where warmer environments may increase the rate of beneficial mutations due to the general positive effects of temperature on biochemical and biophysical processes, extreme environmental conditions may result in the proliferation of deleterious ones (Dandage et al. 2018; Chu et al. 2020; Berger et al. 2021). Interestingly, Berger et al. (2021) reported that the strength of selection on mutational load, that is, the accumulation of deleterious mutations, is not affected by environmental stress per se. Several studies have provided evidence for climate change, especially rising temperatures, to affect mutation rates in various taxa, including bacteria, yeasts, nematodes, and insects (Ogur et al. 1960; Mongold et al. 1999; Matsuba et al. 2013; Muller 1928; Berger et al. 2017). It is therefore not surprising that species have evolved mechanisms to protect their genetic material and associated translatory and epigenetic processes against abiotic stressors, including temperature extremes.

**FIGURE 1:**
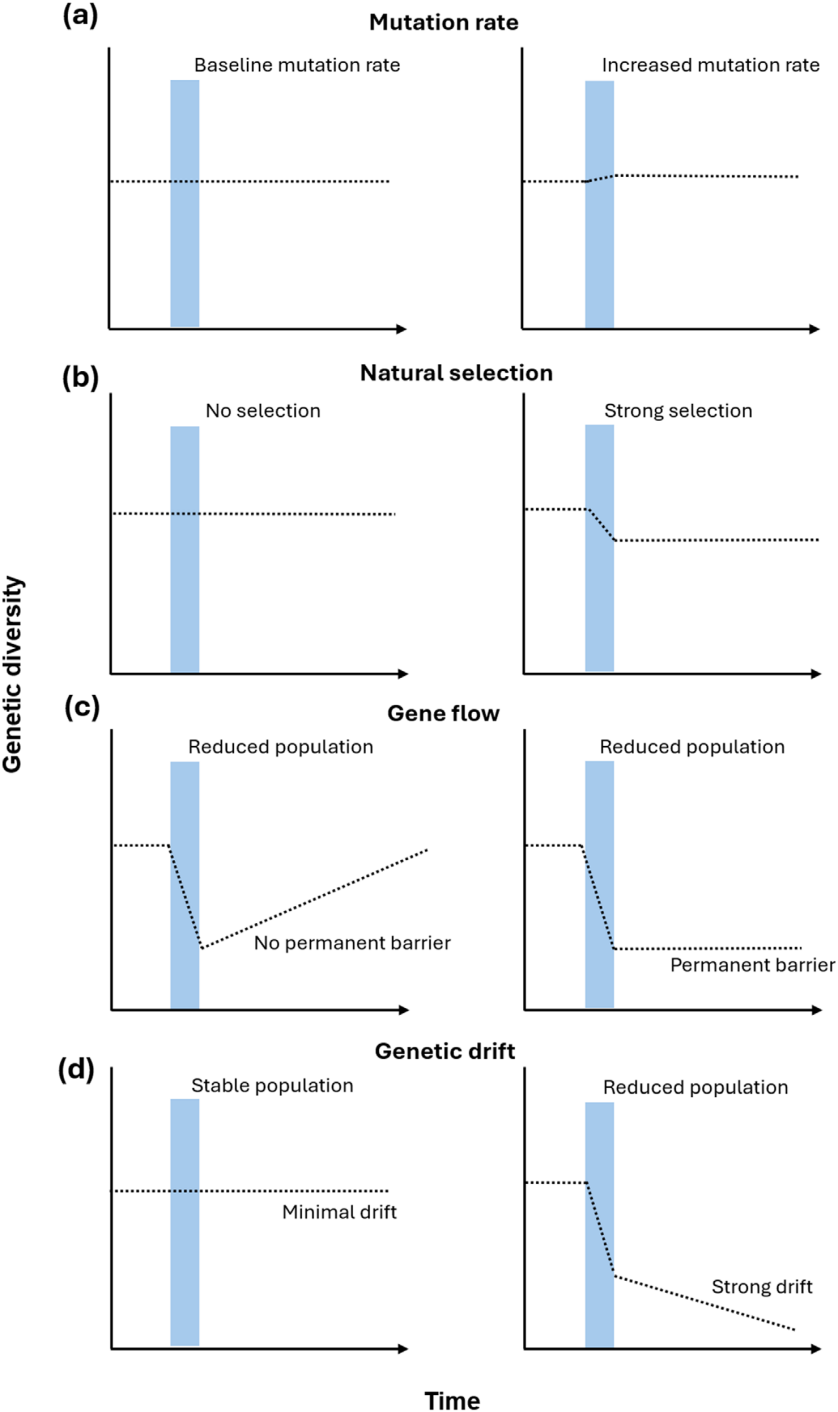
Hypothetical scenarios depicting how extreme climatic events (ECEs) may affect four main drivers of evolution, that is, mutation rate (a), natural selection (b), gene flow (c), and genetic drift (d), with consequences for genetic diversity. The panels illustrate two contrasting scenarios following an ECE: One where the ECE does not impact any of the four drivers, and another where it does—both in directions supported by existing scientific literature (see main text for examples). The *x*‐axis represents time, whereas the *y*‐axis represents genetic diversity. Blue vertical bars represent a hypothetical extreme climatic event. The dashed lines represent the hypothesized effect on genetic diversity.

Genes that code for heat‐shock proteins have been reported in all examined species and can help maintain cellular function and integrity under heat stress (Feder and Hofmann 1999; Chen et al. 2018). Although climatic effects on mutation rates may frequently occur, mutation rates alone are unlikely to contribute strongly to changes in the genetic diversity of populations in the short term (Figure 1a). As most mutations are neutral or deleterious (Bao et al. 2022), it typically requires many generations of recombination for a favorable mutation to arise and become established in a population, and, hence, for meaningful contributions to genetic diversity to be maintained in the population, particularly under stresses imposed by ECEs. Nevertheless, alterations in mutation rates may affect a species' potential for adaptation in the long run after multiple generations, and, thus, exposure of the number of generations of a given species to ECEs could be vital to link their rapid adaptive potential against ECEs.

### 2.2 Natural Selection and Climate Change

Natural selection is the process through which individuals with a given phenotype are better adapted to local conditions, and consequently have higher survival rates, produce more offspring, and hence have higher fitness than those with a less‐adaptive phenotype. Climate has been an important force of natural selection at geological time scales (Siepielski et al. 2017). Periods of extreme change have played an important role in extinctions and following speciation events in earth's geological history. A clear example of this is the Paleocene‐Eocene Thermal Maximum, a relatively brief moment in geological time 56.3 Mya during which the earth's surface temperature warmed by 3–5 degrees centigrade over the span of several thousand years and which was followed by a long period of slow and gradual cooling (Jaramillo et al. 2010). This event coincided with the rise of multiple new lineages of mammals, including ungulates and primates (Gingerich 2006), neotropical angiosperms (Jaramillo et al. 2010), as well as several extinctions that occurred in marine lineages and subsequent migrations to vacated niches by other lineages (Speijer et al. 2012).

Present‐day global biodiversity and geographic distribution patterns are strongly determined by climate attributes, such as mean annual temperature and rainfall and seasonal variations in these parameters (Woodward et al. 2004). Different climatic conditions can select for genotypes that are better adapted to local environmental conditions (Bemmels and Anderson 2019; Johnson et al. 2009). A striking example of this is a reciprocal study with 517 *Arabidopsis thaliana* natural plant lineages that originated from two regions with different temperature and precipitation patterns (Exposito‐Alonso et al. 2019). Grown under hot and dry conditions, lineages that survived and reproduced were exclusively from the warmer and drier regions of origin, suggesting that selection had favored plant genotypes that were better adapted to these conditions. This example illustrates that despite natural selection taking place, under extreme climatic conditions population sizes and genetic diversity can be extremely reduced (after all, only genotypes that survive the ECE until reproduction can contribute to the genetic diversity of future generations, Figure 1b).

### 2.3 Gene Flow and Climate Change

In natural populations, genes move between populations through migration and genetic exchange through reproduction, introducing new genetic variants and increasing genetic diversity of populations. Particularly in small and fragmented populations, gene flow helps to prevent the deleterious effects of inbreeding within a population by maintaining high levels of genetic variation via genetic exchange with neighboring populations (Couvet 2002; Lenormand 2002; Theodorou and Couvet 2006). As gene flow depends on dispersal via migration (Lenormand 2002), the maintenance of gene flow is largely determined by spatial connectivity between populations and the stability of these connections in time (Watts et al. 2015). Climate change complicates this spatial connectivity—not uniformly, but through patchy, localized disruptions, such as those caused by ECEs (Thakur 2020). For instance, ECEs may seriously fragment or eliminate critical habitats within a species' range, while leaving others intact, fracturing populations that were contiguous before into isolated remnants. What follows is not just demographic thinning, but a breakdown in reproductive continuity, where formerly connected groups no longer exchange genes, setting the stage for cumulative genetic erosion (Franks and Weis 2009; Bijlsma and Loeschcke 2012; Rubidge et al. 2012; Duffy and Jacquemyn 2019; Figure 1c). Alternatively, some species may escape local adverse climatic conditions by seeking more favorable niche space or refugia leading over time to range shifts (Hof 2021). Through such range shifts, different parts of the population may segregate, become isolated and reduce the possibility for gene flow. Range shifts may push closely related species to overlap their distributions potentially leading to novel, broadening or shifting hybridization zones, or alternatively pull two overlapping and hybridizing species apart, resulting in the narrowing or removal of hybridization zones (Scriber 2011; Taylor et al. 2014, 2015). Hybridization zones may serve to safeguard genetic diversity and can contribute to speciation (Vallejo‐Marín and Hiscock 2016), but at the same time may be maladaptive and deleterious (Chunco 2014), and hybridized species may also be more vulnerable to ECEs (Hudson et al. 2021; Arce‐Valdés and Sánchez‐Guillén 2022; Bourret et al. 2022). It should be noted that impacts on hybridization events are more plausible under ACC than ECEs, given that the latter are typically short‐lived in nature and survival may be prioritized over reproduction (and hybridization). It is also important to note that even without local extinctions or range shifts, ECEs can render the migration process itself more challenging, for instance through the creation of unfavorable conditions through habitat barriers (i.e., urban expanses or agricultural landscapes), as well as the depletion of essential resources, critically impacting whether and over what distance migration can occur (Maron et al. 2015; Shaw 2016).

### 2.4 Genetic Drift and Climate Change

A large portion of population genetic variation can be attributed to chance. Through genetic drift—a stochastic process rooted in the random transmission of alleles from one generation to the next—allelic variants can disappear entirely from a population, increasing homozygosity and eroding genetic diversity. Although genetic drift operates independently of environmental pressures, its population‐level impacts are magnified under conditions increasingly shaped by climate change. For instance, drift exerts the strongest influence in small populations (Bijlsma and Loeschcke 2012; Jensen and Leigh 2022), and ECEs may act as catalysts that accelerate genetic erosion and population decline. By sharply reducing population sizes, ECEs amplify the impact of drift, potentially pushing species toward extinction vortices. In such cases, the loss of genetic diversity doesn't just reflect past stochasticity—it undermines the population's capacity to adapt to multiple future stressors, including novel or shifting ECE regimes (Figure 1d).

Although often treated as discrete drivers, mutation, natural selection, gene flow, and genetic drift rarely operate in isolation—particularly under the disruptive pulse of ECEs. These events can simultaneously intensify selection pressures, fragment populations, alter dispersal pathways, and even elevate mutation rates through physiological stress, thereby entangling the evolutionary machinery in complex, often unpredictable ways. In the next section, we explore emerging frameworks linking ECEs and evolutionary dynamics, arguing that evolutionary trajectories may be altered more abruptly and profoundly by climate extremes than by gradual shifts in average conditions.

## 3 Resilience—Does What Doesn't Kill You Really Make You Stronger?

Ecological stressors are environmental conditions under which optimal performance and functioning of a species are impaired. Depending on the duration and intensity of exposure to environmental stressors such as ECEs, most organisms can deal with or tolerate these adverse conditions—to some extent (Kingsolver and Buckley 2017; Buckley and Kingsolver 2021). Zhou and Wang (2023) proposed three mechanisms by which the impact of exposure to environmental stress may be limited, or may even be positive. First, through trade‐offs between biological functions (e.g., between reproduction and survival/longevity, Marshall and Sinclair 2010; Lü et al. 2014) negative effects on one trait can be compensated by positive effects in another trait, referred to as ‘see‐saw’ effects. Second, when responses to stressors share regulatory pathways, cross‐tolerance to these stressors can evolve, and as a result, beneficial induced responses to one stressor may also provide protection against a second one (e.g., cold and desiccation, cold and immune stress, Sinclair et al. 2013, and other examples in Rodgers and Gomez Isaza 2023). This can be particularly important to mitigate the effects of multiple stresses associated with a single ECE, such as desiccation and salinity during drought (Pallares et al. 2017) and heat acclimation and hypoxia resistance during heat (Maloyan et al. 2005). A third mechanism deals with mitigation of stress exposure when these events occur temporally separated (Hughes et al. 2019; Rodgers and Gomez Isaza 2021). Such memory or priming effects occur when an organism retains information from previous exposure to a stressor and uses this to deal more efficiently with a second stressor of the same type (referred to as cis‐memory effect) or of a different type (referred to as trans‐memory effect) (Hilker et al. 2016). The stress responses discussed above take place at the level of the individual, but, as the authors argued, consequences at the population level are also expected to be positive when many individuals in a population respond similarly, for instance, if natural selection has favored the occurrence of this adaptation within the population (Zhou and Wang 2023).

When environmental stressors affect population characteristics negatively, often other population characteristics can partly compensate for these negative effects, for instance through demographic shifts, or changes in resource exploitation (Schröder et al. 2009; Reed et al. 2013; Capdevila et al. 2020). An important question is whether such ECE‐induced responses in the short term can mitigate exposure to stressors in individuals, populations and even communities in the long run (Martínez‐De León and Thakur 2024). When environmental stressors are consistent and predictable, it may be possible to evolve adaptive responses. However, particularly when the stressors are unpredictable and variable in nature, which is the case for ECEs (AghaKouchak et al. 2020), the ‘toolbox’ described above to mitigate adverse effects of exposure to (climatic) stressors may not be sufficient to help species withstand them. In addition, standing population genetic diversity severely constrains adaptive responses (Grant and Grant 2002; Reusch and Wood 2007; Smith et al. 2020). Tolerance to environmental stressors associated with ECEs is more likely to evolve when responses to these stressors are mediated by the same regulatory pathway than when different pathways need to interact or act in opposing directions. Most importantly, when environmental stressors affect survival, the genetic diversity of a population will be reduced, which poses a limitation for natural selection to act upon, and, consequently, for new adaptations to evolve (Grant and Grant 2002; Reusch and Wood 2007; Smith et al. 2020).

**FIGURE 2:**
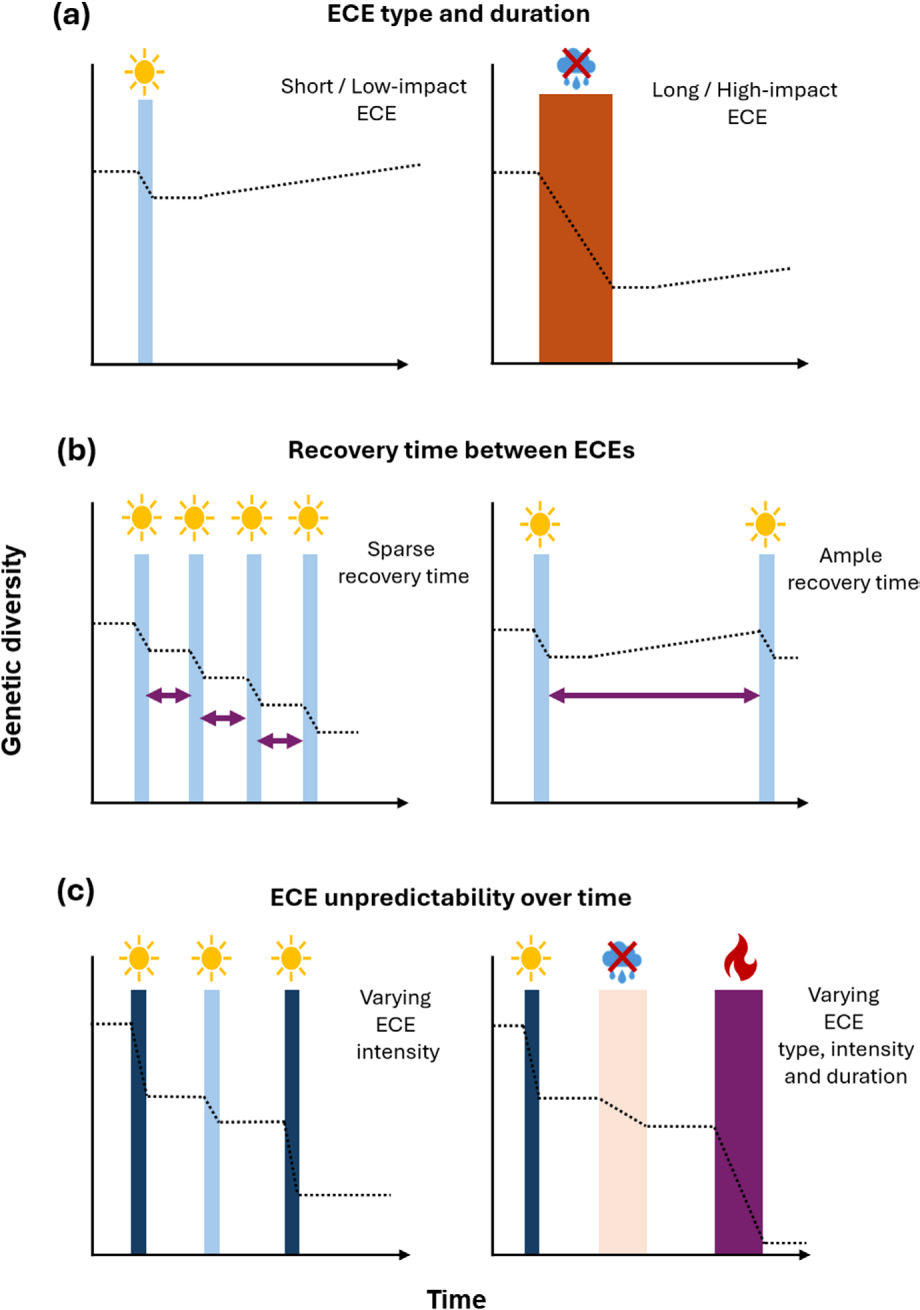
Variability in extreme climatic events (ECEs) in space and time. Panels illustrate the impact of different aspects of ECE variability, and how this is hypothesized to affect genetic diversity and the evolutionary process. The *x*‐axis represents time, whereas the *y*‐axis represents genetic diversity. Colored vertical bars represent a hypothetical ECE. The dashed lines represent the hypothesized effect on genetic diversity. First, (a) ECEs may vary in type and duration (i.e., short temperature ECE on the left, or longer drought events on the right side). Second, (b) ECEs may vary in how often they occur under real‐world circumstances. Some events may follow each other up regularly in a short time period (left side), leaving minimal time for recovery in genetic diversity, for example, through gene flow (short purple arrows). Alternatively, ECEs may occur at longer intervals (right side), allowing for greater recovery time for genetic diversity. Lastly, (c) ECEs are rarely predictable, and may occur in different combinations, intensities and durations, exemplified by repeated temperature ECEs of different intensities (left side), or a succession of multiple ECEs of different types, intensities and durations (right side), likely leading to the strongest reduction in genetic diversity.

Only few papers have reported on the effects of recurrent exposure to ECEs on an organism. In one study that examined the response of coral reefs in the Great Barrier Reef to consecutive heatwaves in 2016 and 2017, coral bleaching was found to be less pronounced following a second, more intense heatwave, which was attributed to memory effects (Hughes et al. 2019). In another study, Anderegg et al. (2020) investigated the effect of subsequent droughts on tree growth and survival. They found that growth rates were reduced more strongly following a second drought, but only when consecutive droughts were severe. Moreover, gymnosperms were more sensitive to consecutive droughts than angiosperms with oak (Fagaceae) even exhibiting acclimation in response to a second period of drought (Anderegg et al. 2020). These studies are examples of immediate responses to repeated ECEs of the same type and do not elucidate longer term impacts of exposure to ECE. The potential to adapt to repeated exposure to ECEs, in particularly when they are not of the same type, is likely reduced when populations are small, when stressor‐caused mortality is high, or when populations are reproductively isolated. This reveals a clear knowledge gap in our understanding of how recurring ECEs, of the same and different type, impact on population health and genetic diversity (Figure 2).

## 4 What Does Not Kill You Could Make You Weaker: Combined Extreme Climatic Events and Evolutionary Bottlenecks

ECEs are characterized by their variability and intensity, which contribute strongly to their unpredictability in several ways (Figure 2). First, extremes can operate at both ends of a continuum for any of the environmental parameters involved (e.g., drought versus flooding for precipitation, cold versus heat for temperature), and extremes on one end may pose completely different challenges to an organism than extremes on the other end of that continuum (Figure 2a, Harvey et al. 2023). Secondly, ECEs may have different impacts, and be perceived very differently, at different times of the year. For instance, extreme temperature increases in summer will have different consequences for organisms than the same temperature increases occurring in winter, as different life stages often have different environmental requirements (Harvey et al. 2020, 2023). Thirdly, climate—ECEs included—is a compound environmental condition, meaning that it is determined by the variation in a range of environmental parameters, including temperature, humidity, precipitation, among many others (Bailey and van de Pol 2016). Extremes in these parameters are often—but not always—correlated. For instance, heat waves, perhaps the most studied of ECEs, often do not only include high temperatures but are frequently accompanied by other extremes, for instance drought or extreme precipitation (AghaKouchak et al. 2020; Jackson et al. 2021; Sanchez‐Mahecha et al. 2024). These conditions may occur concomitantly or in sequence over short periods of time (i.e., hours to days). Alternatively, they may only occur very infrequently, with long periods of time in between (Figure 2b). To make matters worse, ECEs are unpredictable in type, intensity, duration and frequency, which makes them particularly challenging to cope with (Figure 2c). This unpredictability is where the real danger lurks, when considering the effects of climate extremes on evolutionary trajectories, and it is vital that we consider it when we discuss impacts of ECEs or their individual environmental components on populations.

It is without question that ECEs of any kind can push many species' populations over critical thresholds into conditions that do not meet their ecological demands or niche space. For instance, extreme precipitation‐caused floodings or climate‐fueled megafires can rapidly push species outside their tolerance zones, leading to high mortality and the fragmentation of populations (Wintle et al. 2020; Jones 2024), but similar effects are often observed with other ECEs, including heatwaves, droughts, and storms (Stillman 2019; Ummenhofer and Meehl 2017). Strong reductions in population size will greatly reduce that population's genetic diversity, with detrimental consequences for the population's evolutionary adaptability and buffering capacity for future extreme events or even disease (Pauls et al. 2013). In the most extreme forms, such ECEs may cause evolutionary bottlenecks if populations are severely reduced.

It is possible that the surviving proportion of a population that was exposed to an ECE had a selective advantage over the proportion that was eliminated. This could, theoretically, be beneficial in case of a recurring event of the same kind hitting that population in the future. However, the important question to ask is what possible advantage this will offer to a population, if the next extreme event is not of the same, but of another nature? Specifically, will natural selection for tolerance of temperature extremes confer a higher degree of tolerance to other abiotic stressors such as drought, or extreme precipitation and flooding? This is highly unlikely; such a culmination of events is instead more likely to decimate the population and its genetic diversity even further. In fact, populations exposed to ECEs, particularly when following up on each other and resulting in high mortality, are likely to be genetically weakened with an elevated risk of experiencing the detrimental effects of genetic drift and inbreeding depression (Figure 2).

One important component to consider when discussing the effects of climatic extremes on evolutionary trajectories is recovery time. Genetic diversity of the population may eventually recover if an affected population is well‐connected to other populations and experiences high levels of gene flow (Figure 2b). However, migration and gene flow take time to occur. Recovery time may be crucial, even at the individual level. Even if the surviving proportion of a population exposed to ECEs had a selective advantage, this does not automatically mean that the surviving individuals are unscathed by it—‘survival of the fittest’ gives no indication of the health status of the survivors. In fact, it is likely that even among the survivors, ECEs will cause detrimental effects in terms of growth, mobility and reproduction, as has been shown for numerous plant and animal taxa (Thakur et al. 2022). For instance, even under sub‐lethal extreme temperature conditions, fertility is often reduced dramatically in many species, although it has been shown that in some cases this can be restored over time (Walsh et al. 2019). After surviving a serious extreme event of any kind, it is plausible that populations will require substantial recovery time for the population to reach preceding population health levels. This is where the unpredictability of ECEs can pose another problem. When ECEs of the same—or different—nature follow each other up in rapid succession, populations are unlikely to have fully recovered and leave substantial ecological debts (Martínez‐De León and Thakur 2024). Exposing a weakened population to one or several subsequent ECEs may severely deplete genetic diversity and lead to the equivalent of an extinction vortex (Figure 2c). An important question that remains largely unanswered is what the long‐term consequences are of exposing populations to repeated ECEs of different types?

## 5 Evolutionary Rescue in a Changing World—How Far Can It Go?

Rapid evolutionary adaptation may offer one of the most direct pathways for species persistence under increasing climatic extremes, a phenomenon commonly called ‘evolutionary rescue’ (Bell and Gonzalez 2009; Chevin and Lande 2010; Bell 2017). By allowing populations to rapidly adapt to avoid local extinction, evolutionary rescue has garnered considerable attention as climate change intensifies (Hoffmann and Sgrò 2011; Carlson et al. 2014). Indeed, under certain conditions, rapid genetic change can mitigate the severity of ECE impacts, particularly when populations have substantial genetic variation, or experience high rates of gene flow between (sub‐)populations (Carlson et al. 2014). For instance, shifts in *de novo* mutations in *Arabidopsis thaliana* resulted in earlier flowering after a sudden increase of aridness in their environment, which ultimately increased fitness (Fulgione et al. 2022). It has also been suggested that strong selection pressure, alongside demographic and dispersal processes, can facilitate evolutionary rescue in novel environments (Gonzalez et al. 2013; Ashander et al. 2016). Yet, translating these insights into robust predictions remains challenging, given that real‐world systems are rife with ecological complexity, genetic constraints, and compounding anthropogenic stressors, including sequential ECEs (Bell 2013; Azevedo et al. 2024).

Despite some evidence that rapid adaptation can provide short‐term relief from climatic stressors, there are clear limits to how far and how fast this rescue process can go before it reaches both intrinsic and extrinsic barriers (Brady et al. 2019; Klausmeier et al. 2020; Morgan et al. 2020). For instance, genetic correlations among traits and limited beneficial mutations may hinder rescue responses, especially as ECEs become more frequent and intense (Travis et al. 2013; Azevedo et al. 2024; Harvey et al. 2020). Additionally, trade‐offs between adaptation to ECEs and overall fitness in variable environments may undermine long‐term rescue prospects (Shaw and Etterson 2012; Martínez‐De León and Thakur 2024). Hence, while evolutionary rescue provides a crucial framework for understanding potential paths to rapid adaptation and hence the persistence of a species, its success depends on a delicate balance among genetic variation, demographic stability, geographic contexts and ecological interactions—factors that continue to determine species survival and extinction under climate extremes (Hoffmann and Sgrò 2011; Acker et al. 2021; Anstett et al. 2021; Shi and Thakur 2023; Mayekar and Rajpurohit 2024).

## 6 Understanding Impacts of ECEs on Evolution: The Importance of Ecological Context

Numerous studies show that gradual climate warming is driving rapid adaptive responses in organisms that are being expressed through processes like distributional shifts, changes in diel or seasonal patterns of activity, alterations in life cycles, and other life‐history related criteria (Callaghan et al. 2004; Hickling et al. 2006; Botkin et al. 2007; Deutsch et al. 2008; Heino et al. 2009; Norberg et al. 2012; Stein et al. 2013; Hylander et al. 2022). Underpinning these adaptive responses is emerging evidence of a clear genetic fingerprint (Gienapp et al. 2008; Hoffmann and Willi 2008; Hoffmann 2010; Pistevos et al. 2011). Many of these studies have focused on biotic responses to longer‐term ‘incipient’ warming, which, despite being rapid in terms of deep evolutionary and ecological time, is quite different in terms of the immediate threats posed to organisms by exposure to ECEs like heat, drought and flooding (Kellermann and van Heerwaarden 2019). Despite this, exposure to short‐term or immediate threats that are posed by ECEs may also drive evolutionary responses that enable species, populations, and even ecosystems to become more resilient to future ECEs (Sejian et al. 2018; Perez and Aron 2020; Sato et al. 2024; Kong et al. 2025). Indeed, this argument has formed the foundation of a recent study arguing in favor of adaptive radiation as a ‘silver lining’ (Coleman and Wernberg 2020) to what otherwise is perceived to be a profound threat to biodiversity at all levels of organization. There are several vitally important criteria, however, that need to be closely examined in order to test and validate this theory, and once they are scrutinized closely, the flaws inherent in this theory to operate under real‐world conditions become apparent.

Firstly, organisms face a myriad of biotic and abiotic selection pressures, of which climate change and associated ECEs are only one (Pelletier and Coltman 2018). Humans are altering and simplifying the biosphere in numerous ways, which include climate change, habitat loss and fragmentation, the introduction of exotic species into non‐native ecosystems, overharvesting and various forms of pollution (Johnson et al. 2017; Tilman et al. 2017). Optimality theory predicts that the expression in phenotypes is driven by trait‐based responses to numerous selection pressures (Taborsky et al. 2021), leading to trade‐offs in fitness functions like fecundity, longevity, competitive ability, and other parameters (Stearns 1989; Roff et al. 2002). Exposure to a suite of human‐mediated stresses is leading to declines in population sizes, thus reducing the genetic variation that is a prerequisite for adaptation to novel threats.

Secondly, some taxa, such as bats and bees are more sensitive to ECEs and other anthropogenic changes, than many other taxa (O'Shea et al. 2016; Festa et al. 2023; Gonzalez et al. 2024). When they experience extreme conditions, that is, heat, they can experience mass die‐offs that significantly reduce their local or even global populations (Ratnayake et al. 2019). For instance, a single recent heatwave in Australia killed up to a third of the total global population of spectacled flying foxes (Mo et al. 2021; Preece 2025). Similarly, mass mortality was reported in urban populations of two insectivorous bat species *Chaerephon plicatus* and *Taphozous theobaldi* , in response to a single heat wave in Cambodia (Pruvot et al. 2019). Thermal stress has been shown to inflict a range of serious physiological changes on bumblebees, including negative effects on fertility, foraging ability and neural function (Kenna et al. 2021; Gérard et al. 2022; Campion et al. 2023). Repeated exposure to elevated temperatures can also decrease thermal tolerance in bees (Maia‐Silva et al. 2021). The ability of bees to acclimate to sequential heat events is dependent on many factors, but at present the prognosis is pessimistic (Bordier et al. 2017; Gonzalez et al. 2024; Vilchez‐Russell and Rafferty 2024). A combination of the unpredictability, increasing frequency, and intensity of ECEs over time may mean that these organisms will never be able to adapt to them, or that recovery from exposure to one event is disrupted by further exposure to more ECEs.

Thirdly, organisms also vary in their ability to recover after experiencing quite dramatic population declines. Species that reproduce prolifically over only a short time (i.e., many insects and other invertebrates, as well as some vertebrate taxa such as rodents) or which have rapid numerical turnover are potentially more resilient than species with low fecundities and long generation times. For instance, early studies reported that house flies and mosquitoes became resistant to DDT within only 2 years of exposure to this pesticide (Metcalf 1973). The rate at which adaptive mutations can arise within these highly ‘r‐selected’ species is clearly higher than among species exhibiting characteristics of ‘K‐selection’ (Pianka 1970; Boyce 1984). Species at the terminal end of long food chains, such as polar bears, which take several years to reach sexual maturity (under extended parental care), and produce only small numbers of offspring, will clearly be less able to rapidly respond to population declines than more fecund taxa (Atwood et al. 2016; Boonstra et al. 2020). Other factors, such as dispersal ability (Hof et al. 2012; Schloss et al. 2012; Travis et al. 2013), competitive ability (Bocedi et al. 2013; Alexander et al. 2015) and seasonal aspects of life cycles and the genetic constitution of populations (Bradshaw and Holzapfel 2008) are clearly important in better understanding how rapidly and efficiently species and populations will respond evolutionarily to ECEs (Lea et al. 2009; Corte et al. 2018). For instance, wingless organisms or clonal plants are less efficient dispersers than winged organisms or plants that produce wind‐dispersed seeds. If ECEs generate suboptimal conditions locally (i.e., via floods, fires or droughts), then organisms may have to rapidly relocate to new habitats. This will invariably create ‘winners’ and ‘losers’, with the net effect being a reduction in the strength of trophic chains or tightly linked food webs.

In 1974, ecologist Daniel H. Janzen said, ‘what escapes the eye, however, is a much more insidious kind of extinction: the extinction of ecological interactions’ (Janzen 1974). The impact of ECEs thus needs to be considered at different levels of complexity, incorporating multitrophic interactions and food webs, in lieu of paying disproportionate attention to individual or even population‐level responses in organisms.

**FIGURE 3:**
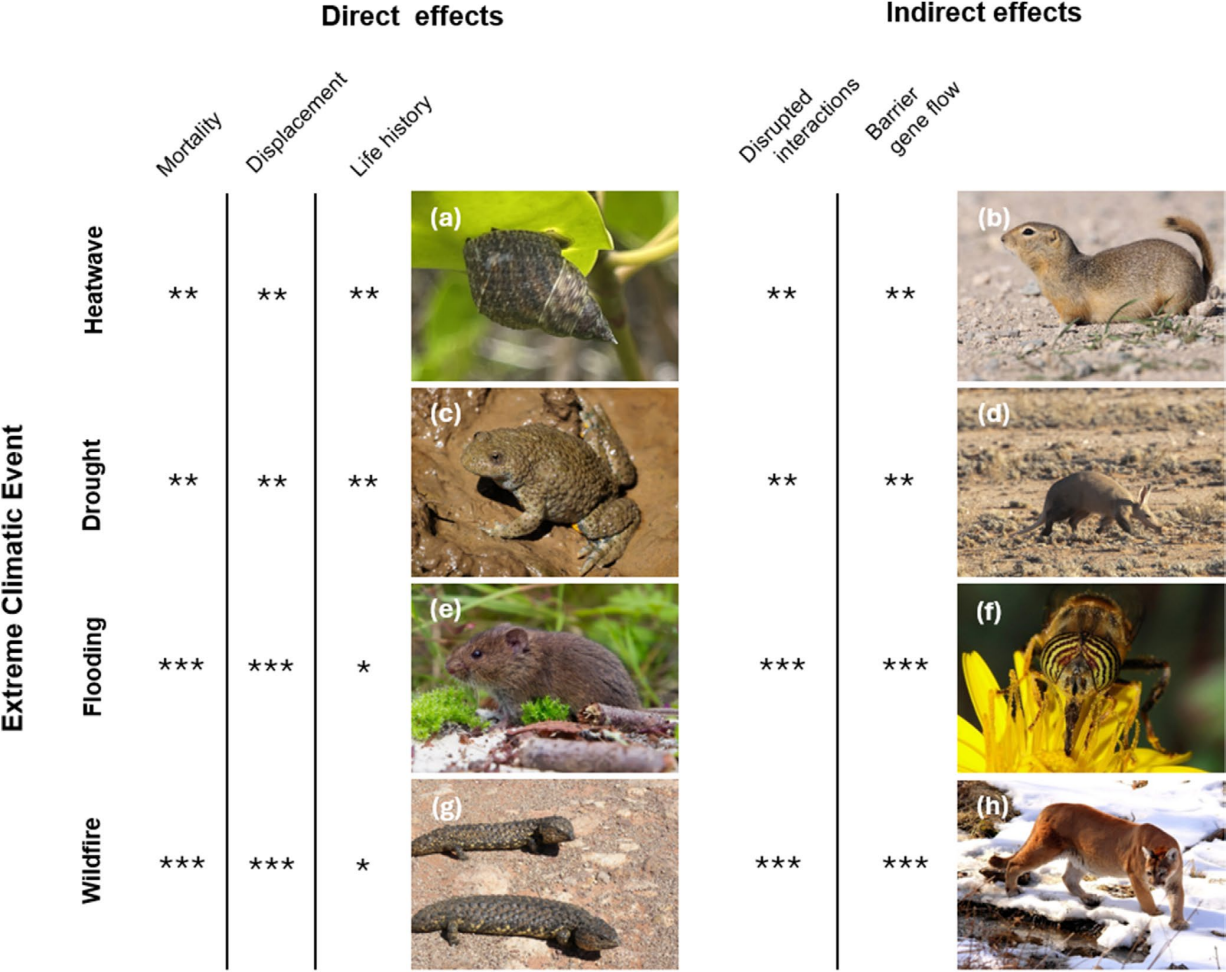
Extreme climatic events (ECEs) may impact species, with consequences for the evolutionary responses in various ways, that can be direct (e.g., mortality, displacement, or effects on life history), or indirect (e.g., disrupted interactions, or lasting barriers for gene flow). Different types of ECEs can have dissimilar direct and indirect impact on species. For instance, heatwaves or droughts tend to build up over time and while often leading to displacement and mortality, are often also mitigated via changes, for example, in behaviour. On the other hand, floodings and wildfires tend to have more immediate effects, with strong mortality or displacement, and are less easily mitigated through behavioral change. Indirect effects are common for most ECEs, for instance via the disruption of trophic interactions, as well as intraspecific interactions. Picture panels show representative examples of species that are reported to have suffered direct or indirect impacts of the respective ECEs: (a) when exposed to high temperatures, the tropical snail *Littoraria scabra* selectively prefers thermally favorable sites, rather than aggregating in specific habitats (Chapperon and Seuront 2011, picture: Joshua Bishop, CC BY), (b) A heatwave during hibernation of Richardson ground squirrel *Urocitellus richardsonii* led to earlier emergence from hibernation in females compared to non‐heatwave years, at a time where the male reproductive apparatus was not yet active, albeit this mismatch between males and females did not affect recruitment (Kucheravy et al. 2021, picture: Hans‐Jörg Hellwig, CC BY 3.0). (c) Yellow‐bellied toads *Bombina variegata* commonly inhabits and breeds in shallow pools, where they are threatened by drought‐related mortality in different life stages (Cayuela et al. 2016, picture: Frank Vassen CC BY 2.0). (d) Aardvarks *Orycteropus afer* are threatened by indirect effects of drought, by starvation, an indication of disrupted trophic interactions (Rey et al. 2017, picture: Niall Perrins CC BY‐NC). (e) Common vole *Microtus arvalis* populations often are completely displaced or killed after floodings, taking substantial recovery time (Jacob 2003, picture: Saxifraga/Rudmer Zwerver—freenatureimages.eu). (f) Pollination events are a common example of interactions that are disrupted by flooding through various mechanisms including accessibility (Nicholson and Egan 2020, picture: Joaquim Alves Gaspar, CC BY‐SA 3.0). (g) Wildfires can be devastating to wildlife, leading to high mortality and displacement. Pineapple skinks *Tiliqua rugosa* show strong sensory responses to smoke and the sound of wildfire, which may enable them to hide or escape from wildfires (Mendyk et al. 2020; Álvarez‐Ruiz et al. 2023, picture: Alan Couch CC BY 2.0). (h) In addition to direct effects on mortality, wildfires indirectly affect Mountain lions *Puma concolor* , via the creation of avoidance behavior towards burnt areas, possible posing barriers to gene flow between populations (Blakey et al. 2022, picture: WikiMedia: NaturesFan1226, CC BY 3.0). Asterisks indicate hypothesized strength of the effect.

Organisms do not live in isolation, but typically share their habitat with many other organisms with which they interact in various ways. Often, other organisms are essential for growth and survival, for example, through provisioning of resources (e.g., flow of energy up the trophic chain), regulation of microhabitat conditions (e.g., cooling effects of plants), or breaking down materials into accessible resources (e.g., soil and gut microbes). Moreover, certain biotic interactions can also pose as environmental stressors themselves, in the form of predation, disease, or competition. It is crucial to also consider these other organisms in relation to the impacts of ECEs. While many ECEs impact organisms directly, by affecting organismal behavior or physiology, in many cases, effects can also be indirect, that is, through shifts in resources, disrupted interactions, or the creation of lasting barriers, many of which involve multiple other species (Figure 3).

**FIGURE 4:**
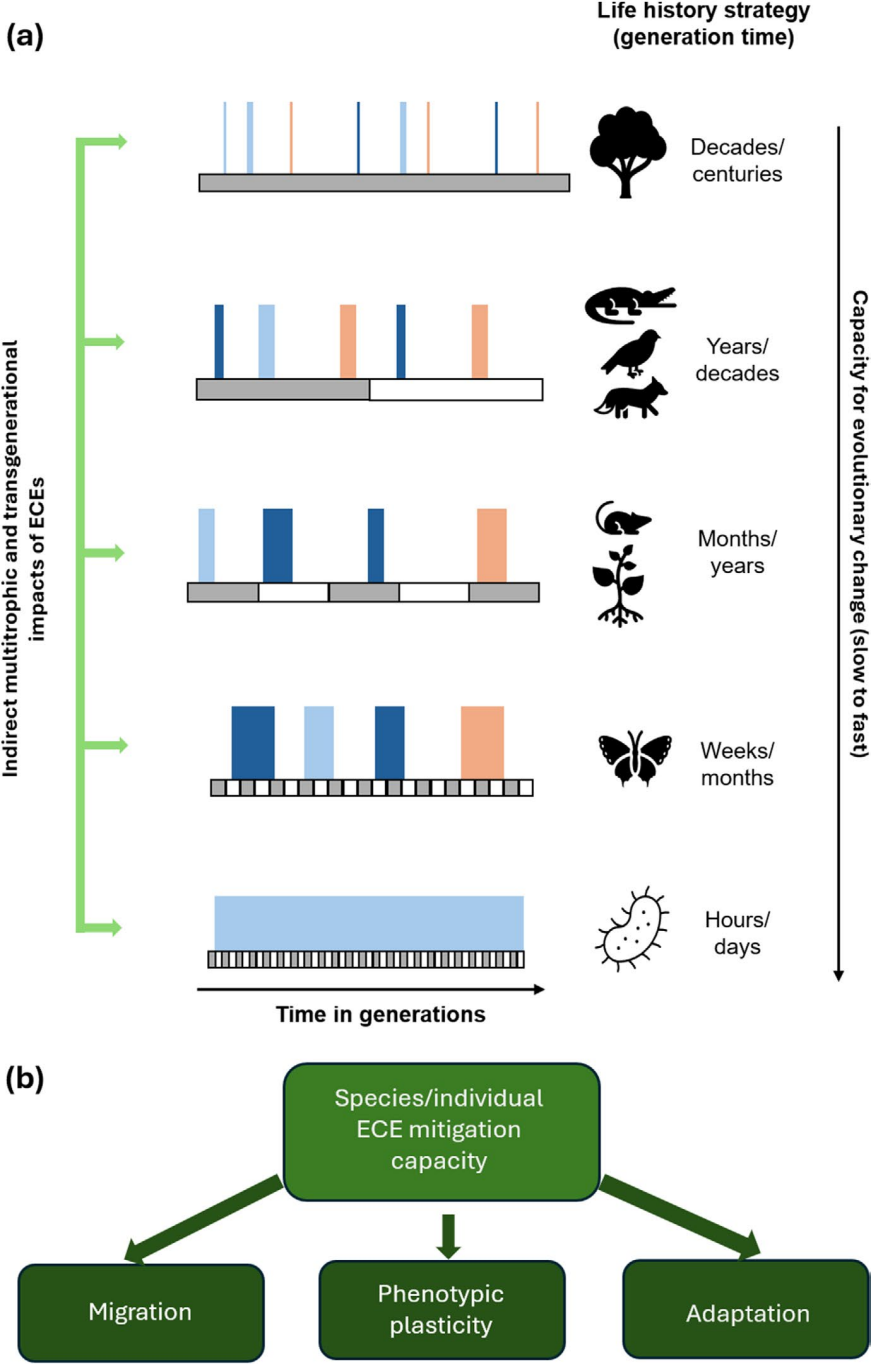
Responses to climatic extreme events (ECEs) in relation to species life history strategies (a). Long‐lived species such as trees can experience several ECEs during their lifetime. These ECEs (vertical bars) can be of the same or a different kind (bar colors), they can differ in intensity (darkness of the bars) and duration (width of the bars). Impact of ECEs differs depending on their duration in relation to the organism's generation time (grey‐white alternating blocks). Generation times at the right are illustrative and hypothetical (e.g., some insects species take several years to complete their lifecycle). Short‐lived organisms may experience only a single ECE in their lifetime (e.g., insects, herbaceous plants, small vertebrates), whereas microorganisms may experience extreme stress conditions over many consecutive generations. All these aspects play a role in whether and how species will respond at both ecological and evolutionary time scales. The timing of these events in relation to an organism's developmental stage may further have consequences for the ability of an organism to respond adequately. This may be in particular the case for insects, which often have complex lifecycles with some life stage requiring even different habitat conditions (aquatic vs. terrestrial). In the figure the first ECE may occur when the insect is in the larval stage, while the second ECE of the same type may occur during the adult stage in the next generation. Granted that species survive a given ECE, they may respond in three main ways (b): They can migrate to areas where conditions are better, phenotypic plasticity may allow species to cope with ECEs to some extent as long as these events are not too extreme, or they can evolve adaptations over time. The response trajectories of species (immediate and long term) are largely determined by species‐specific life history trats, standing genetic variation in a population, and by the trajectories of species with which they interact.

The rate and extent to which organisms respond to environmental stressors resulting in adaptive genetic change is highly variable between species and constrained by life‐history traits (Figure 4). For instance, organisms with long life cycles (e.g., long‐lived trees) are likely exposed to the same stressor, or stressor sequences, multiple times during their lifetimes, whereas short‐lived organisms (e.g., many arthropods) may experience only one stress during their lifetimes, and different generations may experience different types of stress. This will have consequences on how and to what extent species are able to adapt to environmental stressors (Buckley and Kingsolver 2021; Kingsolver and Buckley 2017). In a strongly connected multi‐species network, environmental stressors that strongly affect one species, are likely to also *indirectly* affect other species in the network through cascading effects (Harmon et al. 2009; Gilman et al. 2010; Lavergne et al. 2010; Woodward et al. 2012). An adaptive response to a specific stress in one organism potentially disrupts interactions with other organisms when the latter do not adapt well to the same stress or else might evolve different strategies of coping with it. However, biodiversity may buffer ecosystem functioning against ECEs through functional redundancy (Kong et al. 2025).

A classic example of how interacting species vary in their response to climate change over time is the phenologically‐timed interaction between bud burst of oak trees ( *Quercus robur* ), the peaking of winter moth (*Opheroptera brumata*) abundance and biomass, and reproduction in forest birds. In the Netherlands, warmer springs that occurred in the period between 1975 and 1999 led to progressively earlier hatching of winter moth caterpillars, whereas bud burst in the trees did not advance as rapidly. This resulted in desynchronization between bud burst and development of leaves on which the caterpillars feed (Visser and Holleman 2001). This desynchronization in turn selected for delayed hatching dates in the winter moth (van Asch et al. 2012). This, combined with continued advancement of bud burst between 1995 and 2011, enabled re‐established synchronization of these events (van Asch et al. 2012). In contrast, breeding time of the Great tit ( *Parus major* ) has not advanced to coincide with peak caterpillar biomass that serves as food for their chicks (Reed et al. 2013). Instead, in this species, the fitness costs of the phenological mismatch between food availability and Great tit breeding time are counterbalanced by increased recruitment due to reduced competition (Reed et al. 2013). Thus, interacting species have responded to gradual warming in various ways. However, how the model system above is affected by ECEs, and at which point its functional integrity is lost, has not been investigated.

## 7 Concluding Remarks

The climate of our planet is changing more rapidly now than we have experienced in recent evolutionary history, that is, the Holocene, and poses unique challenges related to the increased pace of change compared to earlier warming events in geological history. Although it is often stated that the planet is warming, increasing surface temperature alone does not capture ACC fully. More accurately, the climate is also becoming increasingly aberrant and less predictable (Day and Hall 2016), with crippling droughts, catastrophic floods and intense megafires frequently making the news in recent years. Natural systems are affected by incipient warming and ECEs, but they pose very different challenges to biodiversity. It should be clear that many populations are suffering from these challenges, and this is likely to become even stronger in the long term. Although gradual warming and ECEs are plausible drivers of natural selection, how this may impact both the micro‐ and macro‐evolution and subsequent genetic diversity is still little known. Whether selection for traits that convey fitness advantages in one ECE scenario will be beneficial in the face of other ECE scenarios is questionable. Whether such a selection will be enough to help threatened species survive is not known, and we suspect it to be less likely. To better understand how ECEs may affect evolutionary trajectories, not only is it crucial to consider populations, communities and ecosystems in their full structural, temporal and spatial complexity, but the impacts of changing climate on genetic diversity need to be closely monitored driven by clear hypotheses. For many taxa, we lack a clear understanding of how ECEs impact population genetics and genetic diversity or evolution at large. Hence, it is pertinent that we work towards a better understanding of how both gradual warming and ECEs impact processes of evolution, through monitoring efforts, or through the generation of population genetics data (e.g., from existing samples from long‐term monitoring efforts). Mounting evidence suggests that, across different levels of biological organization, climate change and associated ECEs are highly destructive and threaten human well‐being through their negative impacts on ecosystems and the vital services they provide. We must not assume that the evolutionary process, however powerful it is, will protect our living planet against the detrimental effects of anthropogenic stressors.

## Author Contributions

**Robin Heinen:** conceptualization, data curation, investigation, methodology, project administration, resources, software, validation, visualization, writing – original draft, writing – review and editing. **Madhav Prakash Thakur:** conceptualization, data curation, funding acquisition, investigation, methodology, resources, validation, writing – original draft, writing – review and editing. **Jeffrey Alan Harvey:** conceptualization, data curation, investigation, methodology, resources, validation, writing – original draft, writing – review and editing. **Rieta Gols:** conceptualization, data curation, investigation, methodology, project administration, resources, validation, visualization, writing – original draft, writing – review and editing.

## Conflicts of Interest

The authors declare no conflicts of interest.

## Data Availability

The authors have nothing to report.
